# Effects of a 4-Week After-School Physical Literacy Program on Health-Related Quality of Life and Symptomatology in Schoolchildren with ADHD: A Study Protocol

**DOI:** 10.3390/healthcare11142113

**Published:** 2023-07-24

**Authors:** José Ignacio Calzada-Rodríguez, María Mendoza-Muñoz, Raquel Pastor-Cisneros, Sabina Barrios-Fernandez, Jorge Carlos-Vivas, Rafael Gómez-Galán, Laura Muñoz-Bermejo

**Affiliations:** 1 Promoting a Healthy Society Research Group (PHeSO), Faculty of Sport Sciences, University of Extremadura, 10003 Caceres, Spain; jocalzada@alumnos.unex.es; 2 Research Group on Physical and Health Literacy and Health-Related Quality of Life (PHYQOL), Faculty of Sport Sciences, University of Extremadura, 10003 Caceres, Spain; 3 Departamento de Desporto e Saúde, Escola de Saúde e Desenvolvimento Humano, Universidade de Évora, 7004-516 Évora, Portugal; 4 Occupation, Participation, Sustainability and Quality of Life (Ability Research Group), Nursing and Occupational Therapy College, University of Extremadura, 10003 Cáceres, Spain; sabinabarrios@unex.es; 5 Physical Activity for Education, Performance and Health (PAEPH) Research Group, Faculty of Sport Sciences, University of Extremadura, 10003 Cáceres, Spain; jorgecv@unex.es; 6 Research Group on Physical and Health Literacy and Health-Related Quality of Life (PHYQOL), University of Extremadura, 06810 Mérida, Spain; rgomez@unex.es; 7 Social Impact and Innovation in Health (InHEALTH), University of Extremadura, 06810 Mérida, Spain; lauramunoz@unex.es

**Keywords:** physical activity, exercise, health, education, neurodevelopmental disorders, attention

## Abstract

Research has shown that physical activity programs led to improvements in children with Attention Deficit Hyperactivity Disorder (ADHD). However, no study evaluating the impact of a physical literacy (PL) program has been conducted. This study aims to examine PL and the effects of an after-school PL program on Health-related quality of life (HRQoL) and ADHD symptomatology including quality and sustained attention. A parallel-group randomised controlled trial will be conducted assessing PL, HRQoL and ADHD symptomatology, both at the beginning and the end of the PL after-school program implementation. The program will last 4 weeks, including two sessions per week lasting 55 min. Sessions will be divided into several parts: greeting (5 min), block I (20 min), block II (20 min) and relaxation and feedback (10 min). Block I will focus on the acquisition of content that contributes to the development of the domains of knowledge and understanding and daily activity; and block II, in addition to favouring physical competence, will seek to improve motivation. If this program proves its effectiveness, it could be an alternative to be included in educational systems, representing a scientific breakthrough regarding physical activity adherence and inactivity-related disease prevention, HRQoL and management of ADHD-associated symptomatology.

## 1. Introduction

Attention Deficit Hyperactivity Disorder (ADHD) is a neurodevelopmental disorder characterised by the presence of persistent patterns of inattention and/or hyperactivity and impulsivity; then, ADHD can interfere with social, academic and occupational functioning [1]. ADHD begins in childhood and can continue into the teen years and adulthood [2]. Whatever the clinical presentation of ADHD (i.e., primarily inattentive, primarily hyperactive/impulsive, mixed) a wide range of negative outcomes related to ADHD in children are recognised, including poorer life satisfaction, quality of life, and lower health outcomes compared to individuals without ADHD [3,4]. Furthermore, they may experience impairments of emotional regulation and social competence, which may compromise their integration with peers and encourage disruptive behaviours. Figures on ADHD prevalence have varied over time, although ranges of 5–7% in children and adolescents are commonly reported [5,6]. Furthermore, as in other neurodevelopmental disorders, their prevalence in boys is significantly higher than in girls, although gender bias in diagnostic criteria has been suggested [7].

Physical literacy (PL) is defined as the motivation, confidence, physical competence, knowledge and understanding to value and participate in a physically active lifestyle [8]. PL includes physical, cognitive, social and affective dimensions measures [9], but it is also a factor that can help to increase Physical Activity (PA) levels (PAL) [10,11]. Moreover, PL is a multidimensional construct which defines an individual’s ability to perform PA moving competently and with attitude, being used regarding physical and mental health throughout life [12]. There is evidence that PL is correlated with different health indicators at school age, including improved health-related quality of life (HRQoL) [9].

PL influence on schoolchildren with ADHD’s HRQoL has not yet been studied. However, several studies indicate that PA may contribute to HRQoL improvement, achieving greater benefits at higher PAL [13,14]. Physical competence is lower in children with ADHD compared to typical children, which may be explained by attention difficulties while acquiring motor skills [15]. Attention problems are a major issue for ADHD, and require proper diagnosis and management [16,17,18]. PA’s benefits have been observed in sustained attention and the quality of attention [19,20,21,22].

Then, considering the PL influence on increased PA, PL may contribute to HRQoL improvement as well as to the quality and sustained attention of schoolchildren with ADHD. There is preliminary evidence that a PA program may improve the cognitive functions and functioning of children with ADHD [23], including attention issues [24,25]. Additionally, PA impacts the mental health of children with ADHD [26], as they show poorer PAL compared to typically developing children [26,27]. Therefore, this study aims to (1) assess the PL level in children and adolescents with ADHD in Spain; and (2) improve PL and consequently HRQoL and ADHD symptomatology, including quality and sustained attention, through a PL program based on PA improvement, motivation, education and healthy lifestyle habits.

## 2. Materials and Methods

### 2.1. Design

A randomized controlled trial employing a parallel-group design will be implemented, including a 4-week intervention phase. Baseline assessments will be administered prior to commencing the intervention, followed by post-intervention evaluations immediately upon its completion. Participants will be randomly assigned to the experimental or control group. The study will be reported following the SPIRIT Statement and the TIDieR Checklist [28,29].

### 2.2. Ethics

The Ethics Committee of the University of Extremadura proceeded to provide the ethical approval needed to develop the present research project, the assigned approval number being 33/2023. In this sense, the study adheres to the revised guidelines of the Declaration of Helsinki, which were amended by the 64th General Assembly of the World Medical Association (Fortaleza, Brazil, 2013) and the Law 14/2007 on Biomedical Research. Furthermore, the study has been properly registered in the Australian New Zealand Clinical Trials Registry under the following Registration Number: ACTRN12623000389606p (accessible at https://www.anzctr.org.au/, accessed on 1 June 2023).

### 2.3. Sample Size

An a priori sample size computation was carried out using the G*Power software 3.1.9.4 (Kiel University, Kiel, Germany), selecting the One-way ANOVA statistical test, that allows the comparison between the means of two independent groups to determine whether there will be evidence that the associated population means will differ significantly. Therefore, considering a significance level of 0.05 (alpha), a 0.02 beta risk, and assuming a large effect size f of 0.4, a total of 52 participants (being 26 subjects included in both experimental and control group) will be sufficient in order to attain a minimum power of 80%.

### 2.4. Randomization and Blinding

Participants will be part of either the experimental (EG) or control group (CG) by following a random assignment process. Prior to participant enrolment (1:1), a randomization sequence will be generated by the free resource Research Randomizer software (version 4.0, Geoffrey C. Urbaniak and Scott Plous, Middletown, CT, USA; accessible at http://www.randomizer.org, accessed on 15 January 2023) [30]. The group assignment process will be conducted by a designated member of the research team who will not have active involvement in the trial. The allocation of participants to either the experimental or control group will be recorded in a computer file secured with a password to ensure confidentiality and data integrity. Neither the outcome assessors nor the data analysts will be aware of the participant’s group assignment.

### 2.5. Participants

In order to be eligible for inclusion in this project, participants must meet the following criteria: (1) having an ADHD clinical diagnosis; (2) no co-morbidities with other neurodevelopmental or psychiatric disorders; (3) aged between 8 and 16 years; (4) not suffering from pathologies that contraindicate the practice of exercise or limit the execution of the PA program; (5) and having authorisation from parents or legal guardians.

### 2.6. Intervention

**Experimental group:** an after-school intervention based on the development of the PL domains will be implemented based on a previous study [31]. Considering the duration of previous PA interventions in this population [32], a period of 4 weeks, with 2 sessions per week of 55 min duration, was established. Sessions will be divided into several parts, starting with a 5 min greeting and exchange of daily experiences, 20 min for block I, 20 min for block II, and 10 min for relaxation and feedback. Aspects such as the adequacy of an attractive playroom favour the acquisition of intrinsic motivation [33].

**Figure 1:**
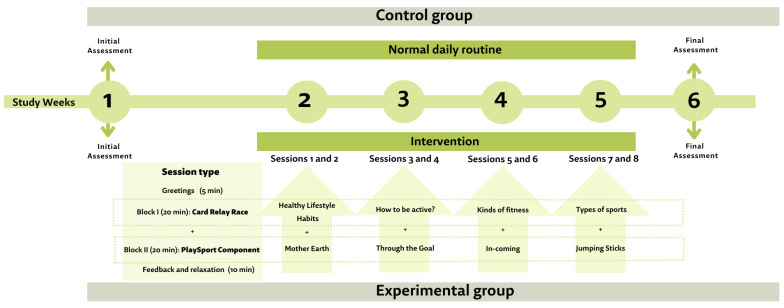
Intervention diagram.

Block I: activities for developing participants’ knowledge and understanding of concepts, as well as attitudes, regarding both healthy lifestyle habits and PA will be carried out through the “card relay race” activity, whereby participants will be organized into teams and positioned in rows. Each member of the team will be provided with a card (model) to be held in his/her hand and will run to a table located about 30 metres away. Once there, he/she will find a series of cards similar to his/her own (stimuli) and will place the card in his/her hand on top of one of the cards on the table that corresponds to his/her own (i.e., look for the stimulus that corresponds to his/her model). Subsequently, he/she will do the race back so that the next partner can come out. Each week, the contents of the activity will be different (healthy lifestyle habits, how to be active?, kinds of fitness and type of sports). Each session, there will be an initial brief explanation of the content to be developed that day (Figure 1). This activity, adapted from a previous study [34] is intended to improve attentional functioning as well as to acquire knowledge about healthy lifestyle habits, while at the same time practising physical activity. Thus, it will contribute to two fundamental pillars of PL such as knowledge and understanding, daily activity and physical competence.

Block II: The second block will be based on active play, essential for values and attitudinal content transmission, as well as on the stimulation of social and civic relationships with others. In line with previous studies on ADHD children [35,36,37], different ball games, including multi-jumping and throwing games, will be performed. Figure 1 shows the games that will be played each week, and their description is provided in PlaySport (http://www.playsport.net, accessed 25 January 2023). The games employed in this study will incorporate elements of both cooperation and competition, as these components have been established to provide motivational benefits [38]. Furthermore, in order to enhance motivation and self-assurance, inclusivity among participants, considering both gender and ability, will be guaranteed by adapting all activities to enable equal learning and active engagement for everybody. Group formation strategies and cooperative activities with a shared objective or purpose will be utilized to promote collective accomplishment [39]. Participants’ active engagement will be ensured by verbal feedback provided by activity coordinators, facilitating their involvement and fostering the development of relationship-infused self-efficacy (RISE) [40,41]. PA and games in children with ADHD can improve perceptual and cognitive functions [42]. Therefore, for the activities that will be developed in the second block, we will try to ensure that they participate in the activities for the mere pleasure of participating and not for a specific result.

For safety reasons, the heart rate will be monitored by the instructor during the session. Participants will be informed at the beginning of the intense efforts about the possibility of raising their hand in case they are not feeling well and need to rest until they recover. Finally, they will be given time at the end of each session to express their opinions [39].

**Control group:** Participants will continue with their usual after-school activities.

### 2.7. Measures

PL and body composition will be evaluated through a variety of tools. Prior to conducting the initial measurements, participants will undergo a familiarization phase designed to acquaint them with the various instruments and assessments incorporated within this study.

Socio-demographic data. Participants’ parents or legal guardians will provide socio-demographic data. The informed consent form will be accompanied by a sociodemographic questionnaire which will include age, gender, type of school (public or private), socioeconomic status, other diagnoses, medication, school year, reinforcement at school, and therapies. These data will only be considered at baseline.

The rest of the measures will be taken at baseline and the end of the intervention and will be carried out by the participants (Figure 1).

(1). Physical Literacy. PL level will be assessed using the Canadian Assessment of Physical Literacy (CAPL-2) [43], from which there is also a preliminary validation in adolescents aged 12 to 16 years old [44]. The CAPL-2 employs a numerical scoring system ranging from 0 to 100 points, with 0 representing the lowest possible value and 100 denoting the highest achievable score. This assessment encompasses four distinct domains: (1) daily physical activity behaviour; (2) physical competence; (3) motivation and confidence; and (4) knowledge and understanding. The different domains will be assessed through a combination of concrete tests and assigned a corresponding score, as outlined below:

a. Daily activity behaviour. The total score for this domain will comprise two components: step counts data derived from an activity wristband (Xiaomi mi Band 3, Xiaomi Corporation, Beijing, China) as a feasible and valid alternative to the research-grade accelerometers [45], which will record the number of steps taken over a full week, and a self-reported inquiry concerning the frequency of engaging in at least 60 min of PA per day. Regarding the total score, it will be determined by combining the score derived from the recorded step count and the score attributed to the individual responses on the weekly PA performed.

b. Physical competence. The scores of the following three components will be combined in order to obtain the ultimate score for this domain:

*I. The Plank test* (30) consists of maintaining the plank position for as long as possible. The total time that the participant held this posture will be taken for the evaluation.

*Ⅱ. Progressive Aerobic Cardiovascular Endurance Run* (PACER) test [46] for assessing cardiorespiratory competence will be used. It involves a 20-m shuttle run (out and back) conducted in response to acoustic signals that define each test-stage intensity. The last stage completed by the participant will be taken for the evaluation.

*Ⅲ. Canadian Agility and Movement Skill Assessment* (CAMSA) [47] will evaluate the participants’ motor skills through employing an agility circuit that comprises various tasks, involving throwing, jumping, and other movements. The time taken to perform the test and the quality of the execution of the movements will be given a score.

c. Motivation and confidence. The CAPL-2 motivation and confidence questionnaire will be used [43]. Thus, participants’ self-confidence in their ability to engage in PA, as well as their motivation towards being physically active, will be evaluated. The scoring will involve the summation of four distinct dimensions, each evaluated on a scale of 1 to 7.5 points: intrinsic motivation, competition, predilection, and appropriateness. Hence, the total score for this domain will range from 1 to 30 points.

d. Knowledge and understanding. Participants’ knowledge about PA will be evaluated in accordance with this domain. [43]. The scoring will be derived from a questionnaire provided in the CAPL-2 manual, which comprises five questions in total. Four of these questions will be in multiple-choice format and scored on a scale from 0 to 1. The fifth question will involve completing missing gaps in a story and will be rated from 1 to 6.

The questionnaires will be filled in the Spanish version [48] following a previous study [49].

(2). Health-Related Quality of Life. It will be assessed using the Child health utility 9D (CHU9D) [50]. The CHU9D comprises a self-report instrument, which must be completed by the child or adolescent, as well as a proxy-report questionnaire completed by the caregiver. It is based on nine items that assess the current state of the child or adolescent across multiple domains, including worry, sadness, pain, tiredness, discomfort, school, sleep, daily routine and activities, reflecting their subjective experience. Each item presents five possible response options, scored on a scale ranging from 1 to 5.

(3). ADHD Symptomatology. The Attention Deficit Hyperactivity Disorder Test (ADHDT) will be used. This tool is designed to identify and assess ADHD from ages 3–23 years [51]. It consists of 36 items in three subscales: (a) hyperactivity, (b) inattention and (c) impulsivity. The total score ranges from 0–72, with higher scores indicating greater and more severe ADHD-related symptomatology.

(4). Quality of attention and sustained attention. It will be assessed based on the Magallanes Scale of Visual Attention (EMAV) [52]. This is used to identify the presence of ADHD and to assess the intensity and impact of the symptomatology in each case. The EMAV scale evaluates the ability to focus attention (Quality of Attention, QA) and to maintain attentional effort over some time (Sustained Attention, SA), as well as stability or performance over a relatively long time. Children should look for and identify figures identical to the model presented. For 8-year-olds, the VAS-1 will be used, where 720 possible figures are presented, of which 140 are identical to the model, and the maximum time to complete the task is 6 min. For children between 9 and 16 years of age, the EMAV-2 will be completed, where 1820 figures are presented, 340 being identical to the model, and the maximum time to complete the task is 12 min. Children’s omissions and errors will be counted to assess their sustained attention and impulsivity, respectively. Their successes are transformed into centiles following the instructions in the scale manual and using the EMAV TIPI-SOFT software [53], to obtain their level of attentional quality.

### 2.8. Statistical Analysis

The Statistical Package for the Social Sciences SPSS (IBM SPSS Statistics, Version 25.0. Armonk, NY, USA) will be used for statistical analyses and computations. Data will be presented as means and standard deviation (SD). The Kolmogorov–Smirnov test and Levene’s test will be applied to check the normality and homogeneity of data. After that, a group × time repeated measures ANOVA will be employed to analyse the impact of the intervention on the various dependent variables. Effect size will also consider Cohen’s d, accompanied by a 95% confidence interval. Statistical significance will be determined for the within-group effect of time and the interaction group × time. Alpha significance level will be established at *p* ≤ 0.05.

## 3. Discussion

PL importance, interest and attention concerning Physical Education (P.E) and PA fields have been raised [54,55,56,57], allowing us to define its framework as an understanding of PA development from a comprehensive point of view and promoting of physically educated individuals. Several studies have evaluated this concept throughout different programs [41,58] which set a physical literacy-based approach and tried to target its domains, carried out by schoolchildren mainly outside mandatory school hours [41,53,58] or either within the P.E subject setting [59,60,61,62]. However, no studies evaluating PL in children and developing specific after-school programs for its improvement in Spain have been found [55]. To our knowledge, this study protocol represents the first PA program focused on improving PL, HRQoL and ADHD symptomatology regarding this specific population of children and adolescents, who experience different difficulties.

The scientific literature has established that worldwide ADHD prevalence among children and adolescents is around 5.7% [6,63], their PAL being usually low and showing risky patterns, such as physical inactivity and sedentary behaviour, which lead to lower quality of life and involve other chronic conditions [64,65]. ADHD youth generally engage in less PA than their non-diagnosed peers and struggle to meet PA recommendations [66] despite the benefits of PA on health and symptoms [67,68,69]. Moreover, higher PAL correlates with greater HRQoL [13] being necessary to improve access to this important health indicator for those with ADHD by enhancing education, knowledge and opportunities for practice. Then, PL represents a useful tool which can lead to higher PAL [10,11] and HRQoL [70]. The proposed intervention would help ADHD children and adolescents meet the PA recommendations for youth, which is to engage in 60 min of moderate to vigorous PA (MVPA) per day at the very least [71]. In this sense, PA stimulates and promotes cognitive and motor skills in the ADHD population [72] and may help to reduce symptoms [73]. As an example of cognitive enhancements, exercise has been proven to benefit not only executive function [74,75,76,77] but attentional processes too [19,20,21,22]. Nevertheless, any previous PL intervention aimed to increase PA within this population has targeted the improvement of attention capacity and its quality as an indirect consequence. According to this, it can be hypothesized that raising PL values may also have benefits regarding this variable.

Under the PL theoretical framework, every individual can be part of the process for developing it regardless of the starting point, so that inclusion is guaranteed even for those children experiencing any issue [78]. Then, PL programs may represent a prospect for enhancing their PAL and raising their health status while feeling competent and integrated, by providing opportunities for participation. The intervention will cover every PL domain by using several strategies promoting motor, psychosocial and cognitive aspects through cooperation and active play. In this sense, activities from the block I, which will include an aerobic component, agree with other studies in which intervallic PA showed several benefits for those with ADHD [42], relay races also being performed within exercise programs as a suitable option for achieving physical, psychological and behavioural improvements, for instance [79,80,81]. Therefore, incorporating relay races into PL interventions may enhance physical and cognitive abilities, while also promoting knowledge and understanding, daily physical activity, and physical competence domains. Likewise, several studies have developed activities similar to those explained for block II of this intervention. The SPARK program included motor movement activities and manipulative motor skills activities, finding improvements in behavioural–cognitive and psychological responses in children with ADHD [37]. Motivational aspects would be relevant when planning and implementing the PL intervention, particularly addressing children’s intrinsic motivation through the distinctive nature of active play, as it could offset the ADHD core symptoms in a game context [82]. Along the same line, teaching games for understanding, skill blocks and active games showed cognitive and affective outcomes related to PL, raising enjoyment towards PA and sport by increasing both participants’ confidence in the instructor’s competence and a sense of self-appreciation for their skills [41]. Accordingly, activities presented in block II, based on different games including balls, multi-jumps and throwing, would be expected to report similar results and increase the engagement in PA.

The study has some limitations, as the daily step counts should be taken with caution because they will be obtained with the Xiaomi mi Band 3 activity wristband (Xiaomi Corporation, Beijing, China) and not with a pedometer as used in the original evaluation [83] or in other studies that used different types of accelerometers [84,85]. However, in this regard, Casado-Robles, Mayorga-Vega, Guijarro-Romero and Viciana [45] have demonstrated good validity between the ActiGraph wGT3X-BT accelerometer used to validate the CAPL-2 in China [85] and the Xiaomi mi Band 3 that will be used in the present study.

Attending to the strengths of this study, assessment has to be highlighted, as CAPL will be used. This instrument represents a comprehensive assessment tool used to evaluate and measure the PL of individuals, particularly children and youth, through evaluating physical competence, daily behaviours, knowledge and understanding, as well as individuals’ motivation and confidence. Furthermore, this instrument is valid and reliable for school children [84,85,86] and adolescents [44], being translated and culturally adapted to the Spanish population [49]. Moreover, HRQoL will be studied by using the CHU9D as a generic preference-based measure of this variable designed for youth which has been already mapped from the KIDSCREEN-10 index [87]. Due to this, PL and HRQoL as linked factors would be perfectly assessed regarding the population of schoolchildren experiencing ADHD, allowing for future initiatives for health promotion and creating contexts that may help them to be physically literate. This study would also establish PL interventions as beneficial for ADHD symptoms and difficulties, showing different pathways and recommendations for caregivers and health professionals in addressing and treating this disorder. Tailored health initiatives designed to promote PL and the overall well-being of young people with ADHD would involve not only targeted and specific interventions, but also educational programs and environmental adaptations which may generate a favourable context for participation in PA and positively impact HRQoL. Then, future research on identifying and creating supportive environments that facilitate the development of PL among schoolchildren with ADHD should be explored, as several factors, such as social support mechanisms and inclusive settings for PA practice, may affect effective engagement and enjoyment towards an active life. In the same line, future work can provide evidence-based recommendations and guidelines which could include strategies for incorporating PL into daily routines and ultimately within the educational curricula and P.E lessons, as well as foster collaboration between the agents involved. Finally, further researches can focus on the potential benefits of PL interventions on symptom management, self-regulation and other specific ADHD manifestations, providing information for health promotion and treatment concerning this particular population, as well as identifying the possible benefits of this concept for other individuals with chronic conditions affecting their well-being.

## 4. Conclusions

The present study will examine the effects of an after-school PL program on HRQoL and ADHD symptomatology in young people with ADHD. Therefore, the main added value of this research will lie in the generation of new knowledge about PL, PA, and ADHD symptomatology, proposing a cost-effective intervention and offering a tool for health promotion and social inclusion for these children and adolescents provided by the educational stakeholders.

If the effects of this programme are demonstrated, it could be very useful for both the public administration and the private sector, as it could offer a low-cost programme that can be easily adjusted to physical condition, age or symptomatology levels.

## Data Availability

Not applicable.
